# 7^th^ Brazilian Guideline of Arterial Hypertension: Chapter 11 - Arterial Hypertension in the elderly

**DOI:** 10.5935/abc.20160161

**Published:** 2016-09

**Authors:** MVB Malachias, S Ferreira Filho, WKSB Souza, JM Ribeiro, RD Miranda, TSV Jardim

Arterial hypertension is the most common chronic noncommunicable disease among the elderly.^1^ Its prevalence increases progressively with aging, AH being considered the major modifiable CVRF in the geriatric population.^2^ From the chronological viewpoint, elderly are individuals aged 65 years and older, living in developed countries, or individuals aged 60 years and older, living in developing countries.^3^ Within that age group, the very elderly are those in their eighth decade of life.^4^

There is a direct and linear relationship between BP and age, the prevalence of AH being greater than 60% in the age group older than 65 years.^5^ The Framingham Study has reported that 90% of the individuals with normal BP levels up to the age of 55 years will develop AH throughout life.^6^ In addition, that study has shown that both SBP and DBP, in both sexes, increase up to the age of 60 years, when DBP begins to decrease. Systolic BP, however, continues to increase linearly.^7^ The high prevalence of other concomitant RFs in the elderly and the consequent increase in the rate of CV events, in addition to the presence of comorbidities, compound the relevance of AH with aging.^8^

Vascular aging is the major aspect related to BP elevation in the elderly, characterized by changes in the microarchitecture of vascular walls, with consequent arterial stiffening. Large vessels, such as the aorta, lose their distensibility, and, although the precise mechanisms are not clear, they primarily involve structural changes in the media layer of the vessels, such as fracture due to elastin fatigue, collagen deposition and calcification, resulting in increased vascular diameter and IMT. Clinically, arterial wall stiffness is expressed as ISH, highly prevalent in the geriatric population, and considered an independent RF for the increase in CV morbidity and mortality.^6,9-11^ Other consequences are increased PWV and elevated PP.^12^

Changes inherent in aging determine different aspects in that population's BP, such as the higher frequency of auscultatory gap, which consists in the disappearance of the Korotkoff sounds during cuff deflation, usually between the end of phase I and beginning of phase II, resulting in falsely low SBP levels or falsely high DBP levels.

The wide BP variability in the elderly throughout 24 hours makes ABPM useful. Pseudohypertension, which is associated with the atherosclerotic process, can be detected by use of Osler's maneuver, that is, the radial artery remains palpable after cuff inflation at least 30 mm Hg above the reading of radial pulse disappearance. The higher occurrence of WCE and orthostatic and postprandial hypotension, and the presence of arrhythmias, such as atrial fibrillation, can hinder BP measurement.^5^

In the elderly, BP should be carefully measured from the technical viewpoint. The recommendations in Chapter 2 should be observed. In addition, it is necessary to assess the presence of postural hypotension, defined as a SBP reduction equal to or greater than 20 mm Hg, or any SBP decrease accompanied by clinical symptoms, and/or a 10-mmHg reduction in DBP when comparing, after 3 minutes, the BP levels obtained in the standing position with those obtained in the decubitus or sitting position.^13^

Previous diagnosis of AH is estimated to occur in 69% of the elderly with previous AMI, in 77% of those with history of stroke, and in 74% of those with history of HF. Although individuals in that age group are more aware of their condition and more frequently undergo treatment than middle-aged hypertensive individuals, the BP control rates among the elderly are lower, especially after the age of 80 years.^6^

In that age group, the treatment of AH has unequivocal benefits in reducing major CV events (AMI, stroke and HF). In addition, there is evidence that it might prevent dementia syndrome, an additional benefit that should be considered in the therapeutic decision.^14-16^

The NPT should be encouraged for all AH stages, based on the adoption of a healthy lifestyle. Although it might be simple and apparently easy to adopt, there is resistance, because it implies changes in old habits.

The main guidance on lifestyle changes that reduces BP and minimizes the CV risk are: physical activity; smoking cessation; loss of excessive body weight; and balanced diet (low-sodium, rich in fruits and vegetables).^15,16^ (GR: I; LE: A). This type of therapy is recommended for the elderly, whose diet is benefited from moderate salt reduction. This lifestyle change is one of the best studied interventions for BP control; the BP reduction is usually more significant when the oldest individuals are considered. The TONE study^17^ provides strong evidence about the effects of dietary sodium reduction for the elderly, with a 4.3-mmHg decrease in SBP and 2-mmHg decrease in DBP of individuals aged 60-80 years with BP < 145/85 mm Hg and daily sodium intake of 5 grams. The benefits of the regular physical activity for the elderly largely extrapolate BP reduction, because it provides better control of other comorbidities, reducing global CV risk. In addition, regular physical activity can reduce the risk of falls and depression, promoting the sensation of general well-being, improving self-esteem and quality of life.^18^

The patients should preferably be accompanied by a multidisciplinary team, and their families should be involved in the entire process, which increases adherence to treatment and its chances of success.^5^

The HYVET study^19^ has shown that active treatment significantly reduces the rates of HF and global mortality in that group. That study has compared active treatment (DIU: indapamide plus, if necessary, ACEI: perindopril) with placebo for octogenarians with initial SBP greater than 160 mm Hg. Target SBP was lower than 150 mm Hg, with a mean BP of 144 mm Hg. A limitation of that important study was that it included elderly usually healthier than the general population.

A large number of randomized studies on the antihypertensive treatment of elderly, including patients aged 80 years and older,^19^ has shown a reduction in CV events due to BP reduction; however, the mean SBP levels attained were never below 140 mm Hg.^20^ Two Japanese studies, comparing strict treatment with mild treatment, have not been able to show any benefit by reducing mean SBP levels to 136 and 137 as compared to 145 and 142, respectively.^21,22^ An analysis of the elderly subgroup in the FEVER study^23^ has shown a reduction in CV events with SBP lowering to below 140 mm Hg, as compared to 145 mm Hg.

There is strong evidence of the benefit of BP reduction with antihypertensive treatment in elderly aged 80 years and older. That advantage is limited to individuals with SBP ≥ 160 mm Hg, whose SBP was reduced to < 150 mm Hg (GR: I; LE: A).

For elderly under the age of 80 years, the antihypertensive treatment should be considered for those with SBP > 140 mm Hg, with target SBP < 140 mm Hg, if they have a good clinical condition and tolerate the treatment well.^19-23^ (GR: IIb; LE: C).

The randomized controlled studies showing the successful effects of antihypertensive treatment on the elderly have used different drug classes. There is evidence favoring DIUs,^12,19,24-27^ CCBs,^28-30^ ACEIs^30^ and ARBs.^31^ The three studies on ISH have used DIUs^12^ or CCBs.^28,29^

A prospective meta-analysis has compared the benefits of different therapeutic regimens for patients divided into two groups by age: under 65 years and 65 years and older. It has confirmed the lack of evidence that different drug classes have different effectiveness in younger or older patients.^32^

It is worth noting the likelihood of secondary AH in the elderly, whose most frequent causes are stenosis of the renal artery, obstructive sleep apnea-hypopnea syndrome (OSAHS), thyroid function changes, and use of drugs that can raise BP.^24,33-35^

Investigating secondary AH in the elderly might be necessary as part of the diagnosis.

Some features of the elderly are worth noting and require a differentiated approach. Elderly with multiple non-CV morbidities, frailty syndrome and/or dementia have an increased risk for functional dependence and death.^36,37^ Despite the trend towards slow BP reduction with the progression of those conditions and organic reserve decrease, some still have significantly high BP levels. Those elderly have not been included in randomized clinical trials, and, thus, should be assessed in an even more global way, carefully weighing the individual priorities and the risk/benefit of antihypertensive treatment, either pharmacological or not. The treatment target should be less strict, with special attention paid to the higher risk of postural and postprandial hypotension. In addition, frail elderly are at higher CV risk, and their treatment should be individualized.

In the presence of established CVD or TOD, they become a priority and should guide both the intensity of treatment, and the choice of drugs.^38-40^ (GR: IIa; LE: C).
